# PROMOTING LIFELONG LEARNING AT OREGON’S AGE-FRIENDLY UNIVERSITIES

**DOI:** 10.1093/geroni/igae098.1681

**Published:** 2024-12-31

**Authors:** Melissa Cannon, Noriko Toyokawa, M Aaron Guest

**Affiliations:** Chair; Co-Chair; Discussant

## Abstract

The Age-Friendly University (AFU) global network “aims to shape how we live and work by increasing educational opportunities across the life span” (https://www.afugn.org/). In Oregon, USA, Portland State University (PSU), Western Oregon University (WOU), and Southern Oregon University (SOU) have endorsed and adopted the AFU Principles to become part of the network of age-friendly higher education institutions. Although each institution has implemented different initiatives as part of this work, a common thread across the three has been promoting lifelong learning. Melissa Cannon will present findings from preliminary research that identified several strengths, weaknesses, and opportunities for WOU becoming age-friendly, including those detailed in their own AFU campus report. Participants will learn some of the recent initiatives that have advanced the university’s age-friendly and lifelong learning efforts. Noriko Toyokawa will guide a discussion on the distinct challenges small-sized universities face when initiating and maintaining age-friendly university initiatives. The presentation will explore potential strategies for effectively managing these situations, using the case of SOU as an illustrative example. Serena Hasworth and Lauren Bouchard will highlight findings from PSU’s “Age-Friendly University Campus Report” and provide an overview of activities to improve age-friendliness through the Better with Age Initiative. They will describe efforts to develop an age-inclusive micro-credential program for students and faculty at PSU. The concluding discussion fosters a dynamic exchange of creative ideas between the audience and presenters, aiming to optimize existing resources for mobilizing and sustaining age-friendly university initiatives across diverse contexts.

